# Sex-Specific Entorhinal Cortex Functional Connectivity in Cognitively Normal Older Adults with Amyloid-β Pathology

**DOI:** 10.1007/s12035-024-04243-z

**Published:** 2024-06-13

**Authors:** Liang Gong, Duan Liu, Bei Zhang, Siyi Yu, Chunhua Xi

**Affiliations:** 1 Department of Neurology, Chengdu Second People’s Hospital, Chengdu, 610017 Sichuan China; 2 Department of Acupuncture & Tuina, Chengdu University of Traditional Chinese Medicine, Chengdu, 610075 Sichuan China; 3 Department of Neurology, The Third Affiliated Hospital of Anhui Medical University, Huaihe Road 390, Heifei, 230061 Anhui China

**Keywords:** Alzheimer’s disease, Sex Difference, Entorhinal cortex, Amyloid-beta, Functional connectivity

## Abstract

Sex and apolipoprotein E (APOE) genotype have been shown to influence the risk and progression of Alzheimer’s disease (AD). However, the impact of these factors on the functional connectivity of the entorhinal cortex (ERC) in clinically unpaired older adults (CUOA) with amyloid-β (Aβ +) pathology remains unclear. A total of 1022 cognitively normal older adults with Aβ + (603 females and 586 APOE ε4 +) from the Anti-Amyloid Treatment in Asymptomatic Alzheimer’s (A4) study were included in this study. The 2 × 2 (gender, 2 APOE genotypes) analysis of covariance was performed to compare the demographic information, cognitive performance, and volumetric MRI data among these groups. Voxel-wise comparisons of bilateral ERC functional connectivity (FC) were conducted, and partial correlation analyses were used to explore the associations between cognitive performance and ERC-FC strength. We found that the APOE genotype influenced ERC functional connectivity mainly in the sensorimotor network (SMN). Males exhibited higher ERC-FC in the salience network (SN), while females displayed higher ERC-FC in the default mode network (DMN), executive control network (ECN), and reward network. The interplay of sex and APOE genotype on ERC-FC was observed in the SMN and cerebellar lobe. The ERC-FC was associated with executive function and memory performance in individuals with CUOA-Aβ + . Our findings provide evidence of sex-specific ERC functional connectivity compensation mechanism in cognitively normal older adults with Aβ + pathology. This study may contribute to a better understanding of the mechanisms underlying the early stages of AD and may help develop personalized interventions in preclinical AD.

## Introduction

Alzheimer’s disease (AD) represents the most prevalent cause of dementia and is characterized by elevated levels of beta-amyloid (Aβ) in the brain [1]. The accumulation of Aβ is a gradual process occurring decades prior to the manifestation of clinical symptoms [2]. Although the early biomarker of Aβ positivity in AD neuropathology has been well-defined, it is believed that other biomarkers, such as structural and functional imaging alterations in conjunction with Aβ + , could contribute to cognitive decline in clinically unpaired older adults (CUOA) with elevated Aβ levels, who are asymptomatic at-risk populations of AD [2–6].

The entorhinal cortex (ERC) is a brain region that plays a crucial role in memory and navigation [7] and is among the initial regions to exhibit pathological changes in AD [8]. Previous studies have indicated that ERC degeneration precedes hippocampal degeneration [9, 10] and that volume loss in the ERC causes a greater functional impact compared to the hippocampus [11]. Furthermore, a recent longitudinal MRI study discovered that Aβ positivity was associated with more significant volumetric declines in the ERC but not in the hippocampus [12]. Based on the dynamic biomarkers model of AD, resting-state functional MRI (rs-fMRI) has been proposed as a tool for investigating biomarkers that temporally precede Aβ amyloid biomarkers [2, 13]. Therefore, exploring the intrinsic functional connectivity of the ERC in CUOA individuals with elevated Aβ could reveal early biomarkers for AD.

Sex differences in cognitive aging and the development of AD have been well-documented in the literature [14, 15]. Previous volumetric MRI studies have shown that males experience greater age-related brain volume change compared to females [16, 17], suggesting that amyloid positivity might exert a stronger impact on males. However, the association between elevated Aβ and early brain changes in CUOA, as well as the role of sex differences in this relationship, remains to be fully elucidated [18]. Recent findings by Armstrong et al. indicate that amyloid positivity correlates with greater ERC volume loss in men but not women [12]. Conversely, recent neuropathological data from post-mortem brain samples by Hu et al. reveal that females exhibit higher hyperphosphorylated Tau hallmarks in the ERC compared to males in cognitively intact elderly individuals, but not Aβ [19]. These findings emphasize the importance of investigating the ERC function in CUOA with elevated Aβ to further understand the intricate interplay between Aβ, sex, and early brain function alteration in preclinical AD.

In this study, we aimed to investigate sex differences in the functional network of the ERC in CUOA with elevated Aβ (CUOA-Aβ +). Considering the sex effects previously found in cognitive aging trajectories, such as faster decline in perceptuomotor speed and visuospatial ability in males and greater resilience to age-related cognitive decline in females [14], we hypothesized that males would exhibit higher ERC functional connectivity (FC) in the temporal and parietal cortex, while females would present higher ERC FC in the frontal cortex in CUOA-Aβ + . Furthermore, we explored the effect of APOE genotype and the interaction between sex and APOE genotype on the ERC-FC network, as APOE ε4 is known to increase the risk and amount of Aβ accumulation [20, 21].

## Methods

### Participants

The present study enrolled participants from the Anti-Amyloid Treatment in Asymptomatic Alzheimer’s Disease (A4) study as of December 20th, 2022. The A4 study is a multi-center clinical trial being conducted in the United States, Canada, Australia, and Japan (ClinicalTrials.gov identifier: NCT02008357). The screening process for the A4 study has been described previously [22]. The inclusion criteria for the current study required that participants were clinically unimpaired older adults (CUOA) with elevated amyloid beta (CUOA-Aβ +) between the ages of 65 and 85. Prior to participation, participants provided written informed consent. Additionally, participants were required to have a Clinical Dementia Rating score of 0, a Mini-Mental State Examination score (MMSE) between 25 and 30, a Logical Memory Delayed Recall (LMDR) score between 6 and 18, and completed amyloid positron emission tomography (PET) scans (^[18F]^florbetapir) and both structural and resting-state functional magnetic resonance imaging (rs-fMRI). The APOE genotype was also obtained for all participants.

### Neuropsychological Test

Cognitive performance was assessed using the Preclinical Alzheimer Cognitive Composite score (PACC) [23, 24], MMSE, Digit Symbol Substitution Test (DSST), Logical Memory Delayed Recall test (LMDR), and Free and Cued Selective Reminding Test (FCSRT, a total 96 scores) [25]. The PACC and MMSE scores assessed overall cognitive performance, while the DSST measured executive function. The LMDR and FCSRT96 evaluated memory performance. All cognitive performance measures were adjusted for age and education in the statistical analyses.

### Neuroimaging Methods

Amyloid ([18F]florbetapir) status was determined by a hybrid quantitative and qualitative method established by the A4 Study [22]. A quantitative mean cortical standardized uptake value ratio threshold of ≥ 1.10 was employed to define amyloid positivity (Aβ +).

Volumetric MRI data were processed using NeuroQuant (NQ), a fully automated segmentation software that has been cleared by the FDA for clinical use (http://www.cortechslabs.com/neuroquant). We selected the bilateral entorhinal cortex volume, and controlled for the effect of the total intracranial volume (ICV).

Detailed rs-fMRI parameters were obtained from the A4 download website (https://ida.loni.usc.edu/pages/access/studyData.jsp?categoryId=126&subCategoryId=238). The rs-fMRI images were preprocessed using SPM12 (http://www.fil.ion.ucl.ac.uk/spm) and DPABI 6.0 (Data Processing & Analysis of Brain Imaging; http://rfmri.org/dpabi) implemented in MATLAB version 8.0 (The MathWorks, Inc., Natick, MA, USA) [26]. The preprocessing includes discarding the first five volumes, slice timing correction, realignment, spatial normalization, resampling (3 × 3 × 3 mm^3^ cubic voxels), low-pass filtering (0.01–0.1 Hz), detrending, regressing out the covariates (24 motion parameters, white matter, cerebrospinal fluid and global mean signals), and smoothing with a 6 mm Gaussian kernel.

Using DPABI, a bilateral, ERC-based, voxel-wise FC analysis was performed. For the construction of the ERC-FC network, the left and right ERC (MNI coordinates: left, − 19, − 12, − 30; right, 19, − 10, − 30) were selected as seed regions from Brainnetome Atlas [27]. The average time course of each entorhinal cortex region was correlated with the time course in all brain voxels using Pearson’s cross correlation, and the correlation coefficients were calculated with Fisher’s Z-transformation to achieve a normal distribution [28, 29]. The mean frame-wise displacement (FD) was applied as a covariate in the imaging analyses. Participants who showed a maximum displacement of > 3 mm and an angular motion of > 3° throughout the resting-state run were removed from the analyses. Moreover, we also used the scrubbing method to censor the bad images with frame displacement (FD) > 0.5, and one image before and two images after the bad image were deleted.

### Statistical Analyses

Statistical analysis was performed using SPSS version 24.0 (SPSS Inc, Chicago, IL) and MATLAB version 8.0. A 2 × 2 (sex, 2 APOE genotypes) analysis of covariance (ANCOVA) followed was performed to compare demographic information, cognitive performance, and volumetric MRI data among these groups. The effect of age and years of education were regressed out in the analyses of cognitive function, while the ICV was regressed out in the ERC volume comparison. Partial correlation analysis was employed to investigate potential associations between cognitive performance and ERC volumes in CUOA-Aβ + individuals, controlling for age, years of education, ICV, and APOE as covariates. Subgroup analyses were also conducted separately for males and females. The statistical threshold was set at *p* < 0.05.

Voxel-wise comparisons of bilateral ERC-FC mapping were conducted using a 2 × 2 (sex × APOE genotype) ANCOVA with age, years of education, ICV, and mean FD as nuisance covariates. Significance thresholds were set at *p* values < 0.005 at the voxel level and *α* values < 0.01 at the cluster level, with voxel sizes above 50, with the Gaussian random field (GRF) correction method for multiple comparisons. The ERC-FC values in the significant brain regions of sex and APOE genotype were extracted for next correlation analyses.

To refine hypotheses regarding the potential functional consequences of the sex special ERC-FC network, the authors used Neurosynth (https://neurovault.org/), a publicly available platform for cognition annotation of brain maps from meta-analysis of neuroimaging literature. The mean spatial correlation coefficients (Pearson’s *r*) between the sex special ERC-FC network and behavioral terms meta-analytic brain activation maps were examined by cross-sample correlation analyses in male and female groups separately. A threshold of |*r*|_mean_ > 0.2 and the top 10 highest significant terms were used for visualization and interpretation. The top 50 behavioral terms associated with sex special maps were also visualized by word cloud maps.

Partial correlation analysis was used to explore the potential associations between cognitive performance, and ERC-FC strength in cognitively normal older adults with Aβ + , age, and years of education were set as covariates. As the right ERC volume was associated with cognitive performance, the volume of the right ERC was also set as a covariate in the correlation analysis between cognitive performance and right ERC-FC value (FDR corrected* p* < 0.05).

## Results

### Sample Characteristics

The sample characteristics were presented in Table 1. This study included 1022 cognitively normal adults with Aβ + (603 females and 586 APOE ε4 +). The mean age, years of education, and intracranial volume (ICV) were higher in males than in females, and the mean age in the APOE ε4 + group was younger than that in the APOE ε3 + group among Aβ + cognitively normal adults. When controlling for age and years of education, females exhibited higher scores in cognitive performance measures, including MMSE, PACC, DSST, and FCSRT96, compared to males in Aβ + cognitively normal individuals. After controlling for age, years of education, and ICV, males had higher bilateral entorhinal volume than females. The APOE ε4 + group demonstrated lower cognitive performance in PACC, FCSRT96, and LMDR than the APOE ε3 + group, when controlling for age and years of education. No significant interactive effect of sex and APOE genotype was found in demographic information, cognitive performance, or volumetric MRI.

**Table 1:** Demographic and neuropsychological data

	Male (*n* = 419)			Female (*n* = 603)			APOE ε3 + (*n* = 436)	APOE ε4 + (*n* = 586)
	APOE ε3 + (*n* = 179)	APOE ε4 + (*n* = 240)	ALL	APOE ε3 + (*n* = 257)	APOE ε4 + (*n* = 346)	ALL		
Age(yrs)	73.48 ± 5.19	72.30 ± 4.80	72.80 ± 5.00	72.48 ± 4.96	70.58 ± 4.12	71.38 ± 4.59^a^	72.89 ± 5.08	71.28 ± 4.49^b^
Education (yrs)	16.79 ± 3.11	17.17 ± 2.64	17.00 ± 2.85	16.12 ± 2.81	16.25 ± 2.64	16.20 ± 2.71^a^	16.39 ± 2.95	16.63 ± 2.68
PACC	− 1.19 ± 2.59	− 1.15 ± 2.62	− 1.17 ± 2.60	0.22 ± 2.67	0.05 ± 2.59	0.12 ± 2.62^a^	− 0.36 ± 2.72	− 0.44 ± 2.67^b^
DSST	40.13 ± 7.65	40.97 ± 8.29	40.61 ± 8.02	43.38 ± 9.25	44.80 ± 9.18	44.20 ± 9.22^a^	42.04 ± 8.76	43.23 ± 9.02
FCSRT96	73.75 ± 6.08	73.13 ± 5.98	73.39 ± 6.03	77.36 ± 5.49	76.77 ± 5.96	77.02 ± 5.77^a^	75.88 ± 6.01	75.28 ± 6.23^b^
LMDR	11.46 ± 3.43	11.31 ± 3.23	11.38 ± 3.32	11.64 ± 3.48	11.28 ± 3.20	11.43 ± 3.32	11.56 ± 3.46	11.29 ± 3.21^b^
MMSE	28.48 ± 1.38	28.57 ± 1.36	28.53 ± 1.37	28.95 ± 1.16	28.80 ± 1.21	28.87 ± 1.19^a^	28.76 ± 1.27	28.71 ± 1.27
ICV (ml)	1632.56 ± 125.65	1637.93 ± 115.75	1635.64 ± 119.95	1436.97 ± 109.96	1451.99 ± 103.80	1445.62 ± 106.62^a^	1517.37 ± 151.20	1528.15 ± 142.14
Left entorhinal (ml)	3.10 ± 0.67	3.10 ± 0.63	3.10 ± 0.65	2.63 ± 0.57	2.63 ± 0.58	2.63 ± 0.57^a^	2.82 ± 0.65	2.82 ± 0.64
Right entorhinal (ml)	2.90 ± 0.64	2.95 ± 0.62	2.93 ± 0.63	2.33 ± 0.52	2.36 ± 0.55	2.35 ± 0.54^a^	2.57 ± 0.64	2.60 ± 0.65

*APOE* apolipoprotein E; *PACC* preclinical Alzheimer’s cognitive composite; *DSST* Digit Symbol Substitution Test; *FCSRT96* free (FR) and cued (CR) selective reminding test, 2*FR + CR; *LMDR* logical memory delay recall; *MMSE* mini-mental state examination; *ICV* intracranial volume

^a^The significant effect of sex, *p* < 0.05

^b^The significant of APOE, *p* < 0.05

The right ERC volume was significant positively associated LMDR score (*r* = 0.08, *p* = 0.007) and PACC scores (*r* = 0.10, *p* = 0.001), controlled the effect of age, education, ICV, and APOE genotype. In addition, the subgroup analysis revealed that this association just significant in the females (LMDR, *r* = 0.11, *p* = 0.007; PACC, *r* = 0.11, *p* = 0.005) but not males (LMDR, *r* = 0.08, *p* = 0.108; PACC, *r* = 0.09, *p* = 0.06) with CUOA-Aβ + .

### APOE Genotype Effect on the ERC-FC Network

**Fig. 1:**
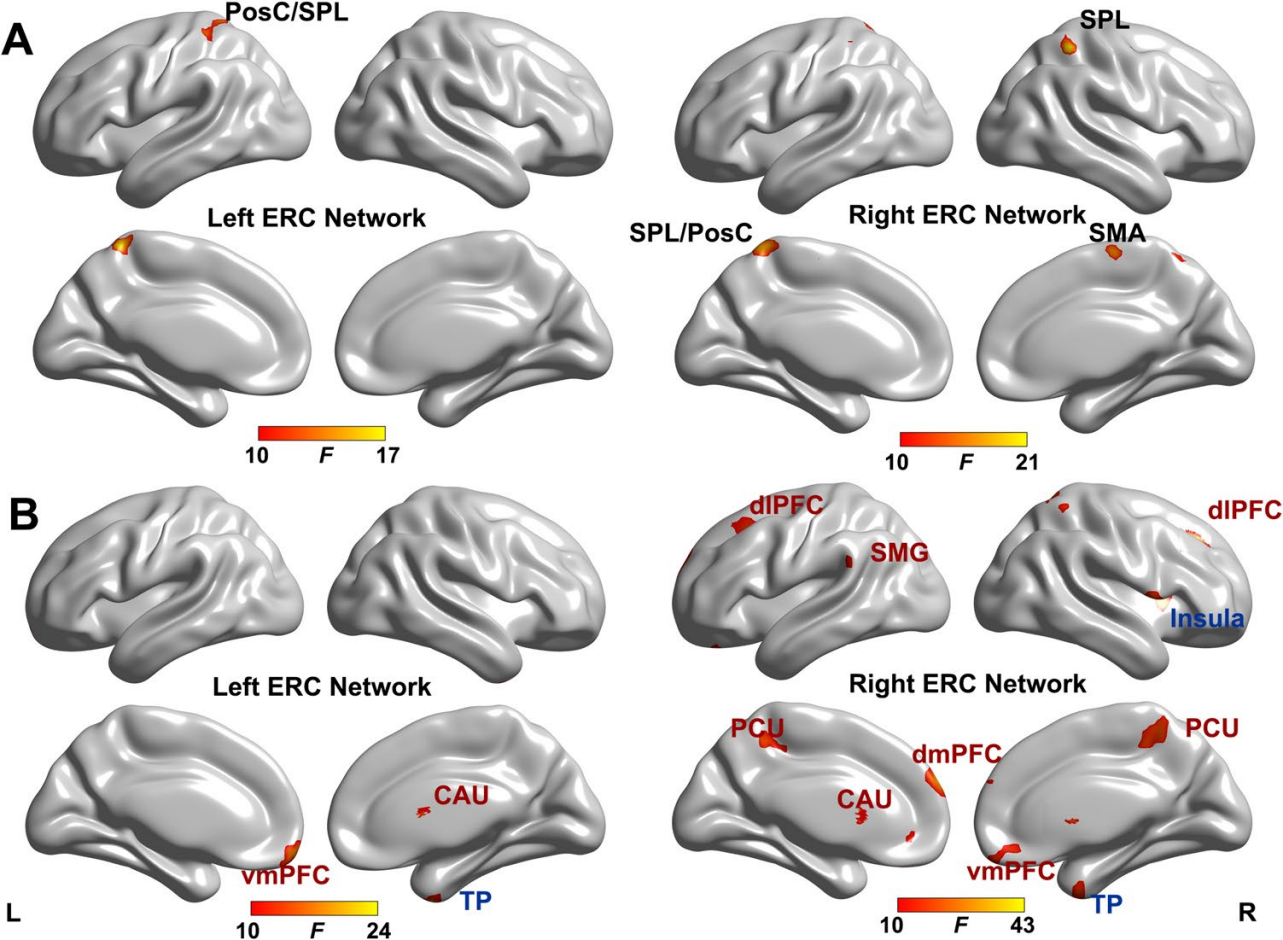
The main effect of sex and *APOE* genotype on ERC-FC network (*p* < 0.005, *α* < 0.01, GRF correction). The brain regions show the significant *APOE* genotype effect (**A**) and sex effect (**B**) on the ERC-FC network. For the sex effect on the ERC-FC network, the blue text indicates that male showed higher ERC-FC, while red text indicates that female showed higher ERC-FC. Color bar means the *F* value. Abbreviations: ERC-FC, entorhinal cortex functional connectivity; vmPFC, ventromedial prefrontal cortex; TP, temporal pole; PosC, postcentral gyrus; SPL, superior parietal lobule; dmPFC, dorsomedial prefrontal cortex; dlPFC, dorsolateral prefrontal cortex; SMG, supramarginal gyrus; PCU, precuneus; PreC, precentral gyrus; SMA, supplemental area

As shown in Fig. 1A and Table 2, the main effect of APOE genotype was found in the left ERC- FC with left postcentral gyrus (PosC) and superior parietal lobule (SPL). For the right ERC-FC network, the main effect of APOE genotype was located in the left PosC/SPL, right SPL, right precentral gyrus (PreC), and right supplementary motor area (SMA). Interestingly, these regions are all located within the sensorimotor network (SMN) [30].

**Table 2:** Brain areas with significant Sex, *APOE* and Sex × *APOE* effects on ERC-FC network

	Brain regions	Brodmann area	Cluster size (voxels)	MNI coordinates (*x*, *y*, *z*)	Peak *F*-score
Left ERC − FC network					
Main effect of sex	Right caudate	–	43	12, 15, 12	15.35
	Left vmPFC	11	79	− 6, 45, − 33	22.33
	Right TP	20	53	36, 6, − 51	24.29
Main effect of *APOE*	Left PosC/SPL	2/5	158	− 9, − 48, 66	17.06
Interactive effect of sex × *APOE*	Left PosC/SPL	3/40	58	− 33, − 39, 42	12.51
Right ERC-FC network					
Main effect of sex	Right vmPFC	11	266	0, 54, − 27	26.43
	Bilateral dmPFC	10	221	− 6, 60, 27	43.28
	Left dlPFC	46	160	− 27, 21, 45	22.12
	Right dlPFC	9	75	27, 27, 42	22.13
	Right insula	48	195	39, 12, 6	26.53
	Bilateral caudate	–	153	12, 15, 9	28.53
	Left SMG	48	91	− 66, − 6, 18	20.12
	Bilateral PCU	7	399	18, − 57, 72	25,17
	Right TP	20	99	33, 3, − 48	23.78
Main effect of *APOE*	Right PreC	6	61	33, − 9, 48	19.74
	Right SPL	40	74	36, − 48, 57	20.73
	Left SPL/PosC	5	229	− 6, − 45, 69	18.67
	Right SMA	4	65	6, − 15, 66	17.18
Interactive effect of sex × *APOE*	Left pCbl	−	39	− 27, − 78, − 39	19.85

*ERC-FC* entorhinal cortex functional connectivity, *vmPFC* ventromedial prefrontal cortex, *TP* temporal pole, *PosC* postcentral gyrus, *SPL* superior parietal lobule, *dmPFC* dorsomedial prefrontal cortex, *dlPFC* dorsolateral prefrontal cortex, *SMG* supramarginal gyrus, *PCU* precuneus, *PreC* precentral gyrus, *SMA* supplementary motor area, *pCbl* posterior cerebellar lobe

### Sex Effect on the ERC-FC Network

The main effect of sex on the ERC-FC network was presented in Fig. 1B and Table 2. For the left ERC-FC network, the sex effect brain regions were located in the right caudate, left ventromedial prefrontal cortex (vmPFC), and right temporal pole (TP). For the right ERC-FC network, the sex-affected brain regions included bilateral dorsomedial prefrontal cortex (dmPFC), bilateral dorsolateral prefrontal cortex (dlPFC), bilateral caudate, bilateral precuneus, right vmPFC, left supramarginal gyrus (SMG), and right insula. The post hoc analysis indicated that males exhibited higher ERC-FC in salience network (SN, insula, and TP), while female displayed higher ERC-FC in default mode network (DMN, precuneus and SMG) and executive control network (ECN, dlFPC), and reward network (vmPFC and caudate).

### Functional Annotation of Sex-Specific ERC-FC Network

**Fig. 2:**
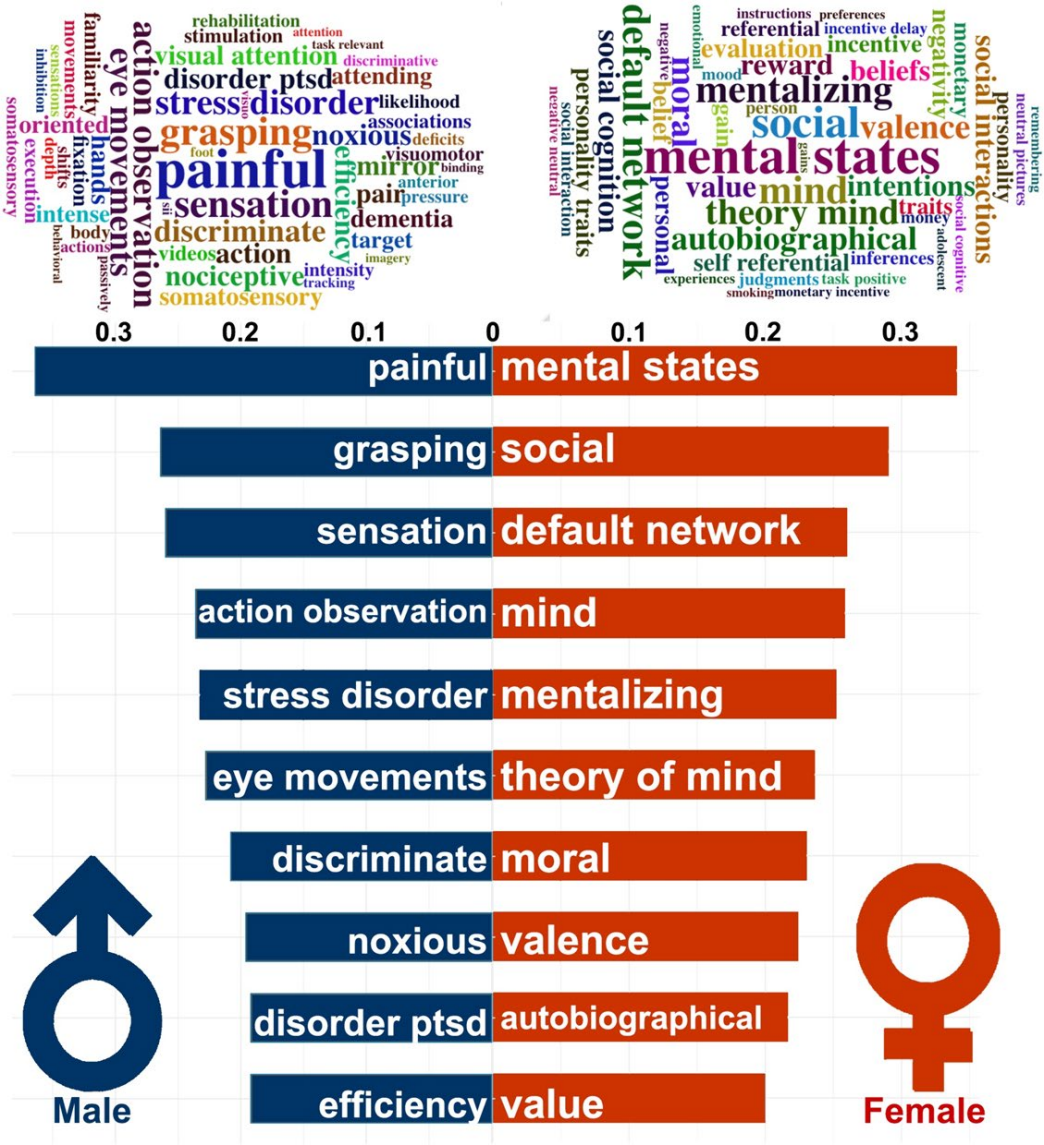
Functional annotation of Sex special ERC-FC network. Up, the word cloud of the top 50 terms associated with sex special ERC-FC network. The font sizes of the terms represented the correlation coefficients. Down, the top 10 highest significant terms associated with sex special ERC-FC maps. The coordinate values represent correlation coefficients between sex special ERC-FC maps and activation values of behavioral terms from the Neurosynth

The study also performed functional annotation of sex-specific ERC-FC network using Neurosynth. We found that the male-specific ERC-FC network in Aβ + group correlated with shared behavioral terms including “painful,” “grasping,” “sensation,” “action observation,” “stress disorder,” “eye movements,” “discriminate,” “noxious,” “disorder ptsd,” and “efficiency,” while the female-specific ERC-FC network in Aβ + group correlated with shared behavioral terms including “mental states,” “social,” “default network,” “mind,” “mentalizing,” “theory of mind,” “moral,” “valence,” “autobiographical,” and “value” (Fig. 2).

### Interactive Effect of Sex and APOE Genotype on the ERC-FC Network

**Fig. 3:**
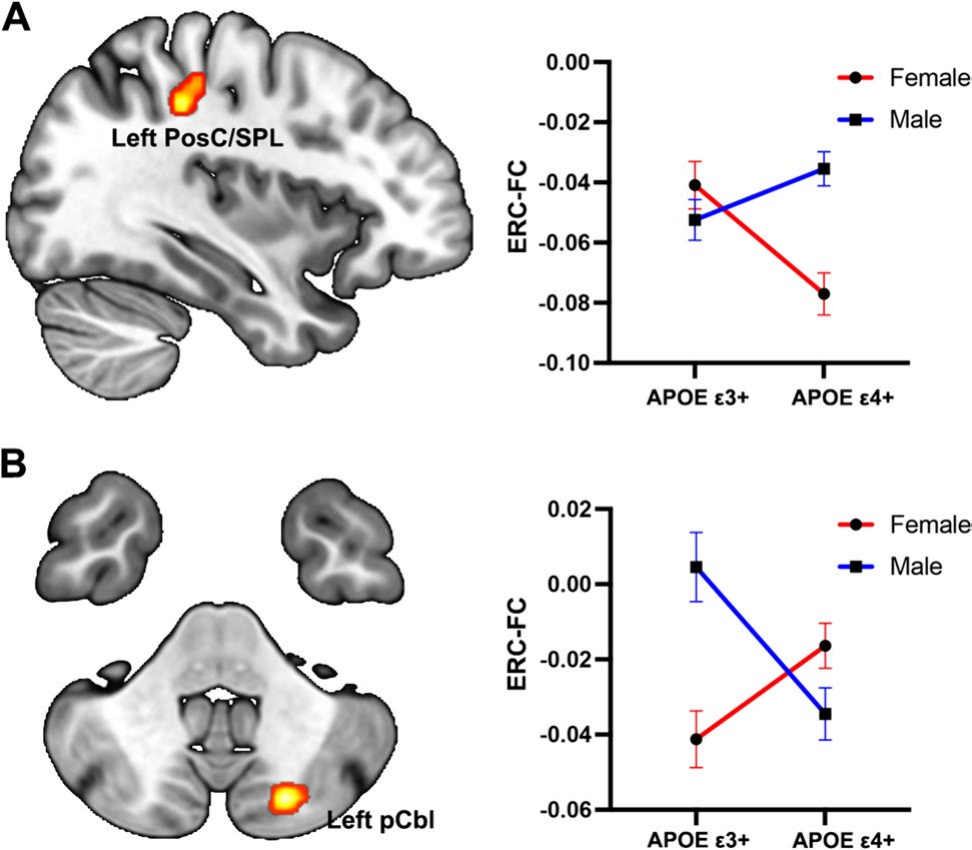
The interactive effect of *APOE* genotype and sex on the ERC-FC network (*p* < 0.005, *α* < 0.01, GFR corrected).** A** For the left ERC-FC network, the APOE ε4 + individuals exhibited greater sex difference on left PoSc/SPL. **B** For the right ERC-FC network, the APOE ε3 + individuals exhibited greater sex difference on the left pCbl. Color bar means the *F* value. Abbreviations: ERC-FC, entorhinal cortex functional connectivity; PosC, postcentral gyrus; SPL, superior parietal lobule; pCbl, posterior cerebellar lobe

As shown in Fig. 3**,** for the left ERC-FC network, a significant interactive effect of Sex and APOE genotype was found in left PosC/SPL. Compared to APOE ε3 + , the APOE ε4 + individuals exhibited higher ERC-FC values in males, while showing lower left ERC-FC value in females. For the right ERC-FC network, the significant interactive effect of Sex and APOE genotype was observed in left posterior cerebellar lobe (pCbl); APOE ε4 + individuals demonstrated lower ERC-pCbl FC in males, while displaying higher ERC-pCbl FC in females.

### Relationships Between ERC-FC Network and Cognitive Performance in Aβ + Preclinical AD

**Fig. 4:**
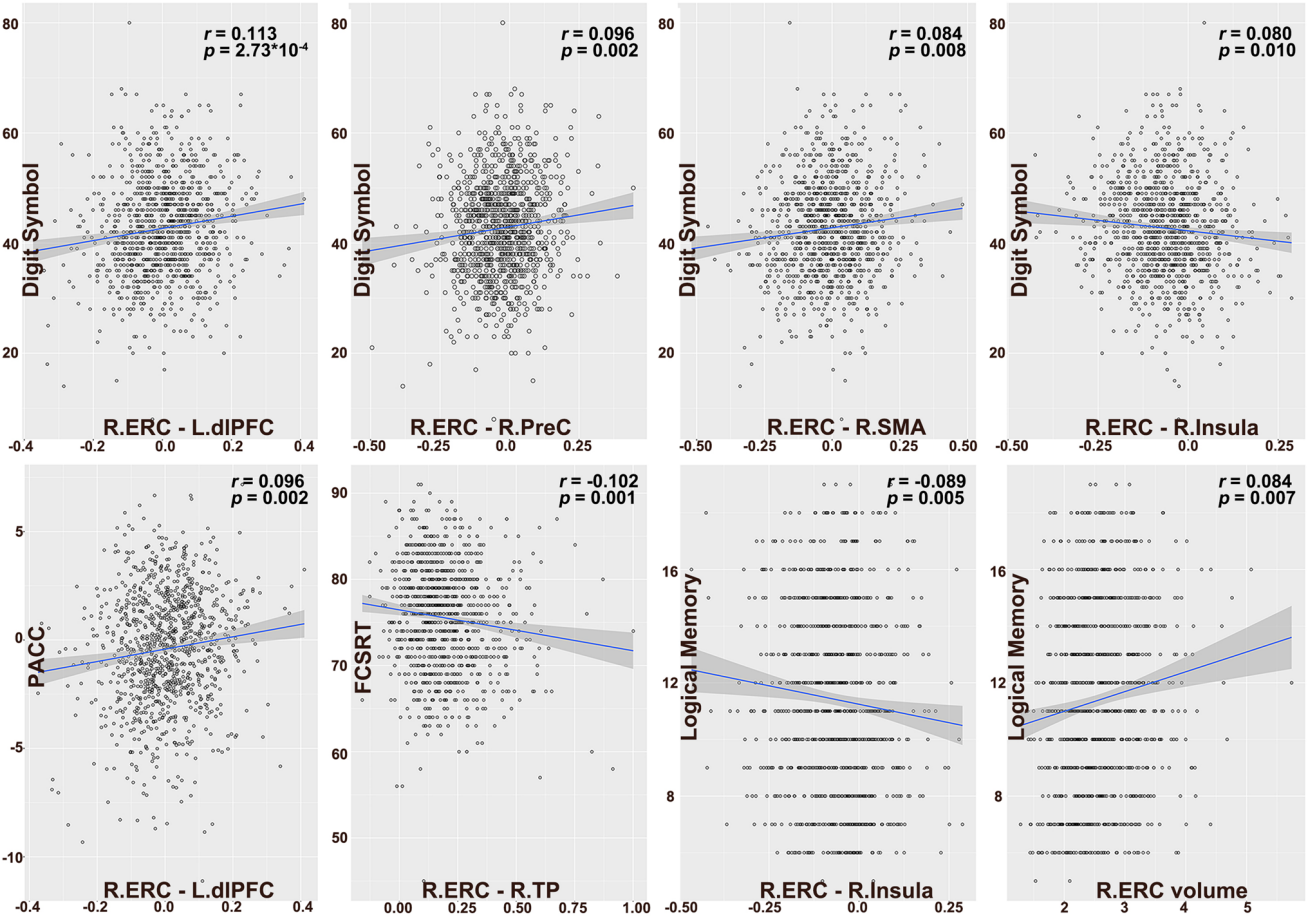
The relationships between ERC volume/ERC-FC and cognitive performance in Aβ positive older. The partial correlation analyses showed that the cognitive performance was significantly associated with the right ERC-FC network, including dlPFC, PreC, SMA insula and TP, and the right ERC volume. Abbreviations: ERC-FC, entorhinal cortex functional connectivity; PACC, preclinical Alzheimer’s cognitive composite; FCSRT, free and cued selective reminding test; R, right hemisphere; L, left hemisphere; TP, temporal pole; PosC, postcentral gyrus; dlPFC, dorsolateral prefrontal cortex; PreC, precentral gyrus; SMA, supplemental area

The partial correlation analyses showed that cognitive performance was significantly associated with several regions of the ERC-FC network. In the Aβ + preclinical AD group, DSST performance was significantly associated with right ERC–left dlPFC FC, right ERC–right PreC FC, right ERC-right SMA FC, and right ERC–right insula FC. The PACC score was significantly associated with the FC between right ERC and left dlPFC, the FCSRT96 score was significant associated with the FC between right ERC and TP. The LMDR score was significant associated with right ERC–right insula FC (Fig. 4). No other significant associations were found between cognitive performance and ERC-FC network in Aβ + preclinical AD.

## Discussion

The current study aimed to investigate the effects of sex and APOE genotype on the functional connectivity of the ERC in cognitively normal individuals with amyloid-β (Aβ +) pathology. Our findings revealed that the APOE genotype mainly influenced the ERC-FC located in the SMN. Males exhibited higher ERC-FC in the SN, while females displayed higher ERC-FC in the DMN, ECN, and reward network. The interplay of sex and APOE genotype was found in the Pos/SPL and left pCbl. The influenced ERC-FC was associated with executive function and memory performance in CUOA-Aβ + individuals. The findings of this study may help to better understand the mechanisms underlying the sex differences in the early stages of AD and may contribute to the development of personalized interventions to prevent or delay cognitive decline in preclinical AD.

Our results align with previous studies that have reported females demonstrating higher cognitive performance in the early stages of AD [31, 32]. Interestingly, despite females displaying lower ERC volumes compared to males, cognitive performance was better in females with CUOA-Aβ + . We also observed a positive association between cognitive performance and right ERC volumes, particularly in females. This might indicate a maladaptive mechanism of ERC structure in females with CUOA-Aβ + [33, 34]. The relationship between ERC volumes and cognitive performance in females suggests that there may be compensatory mechanisms at play, allowing for better cognitive outcomes despite lower ERC volumes [35, 36].

Our findings also revealed that the APOE genotype influenced the ERC-FC in the SMN. This is in line with previous studies, which have reported that APOE ε4 carriers show altered functional connectivity in various brain networks, including the SMN [37, 38]. The SMN has been implicated in the processing of sensory and motor information and is known to be affected in the early stages of AD [39]. The observed differences in ERC-FC between APOE ε4 carriers and non-carriers may reflect early changes in functional brain organization associated with AD pathology, which may eventually lead to the cognitive decline observed in APOE ε4 carriers [40, 41].

In terms of sex differences in the ERC functional network, our results showed that males exhibited higher ERC-FC in the SN, while females displayed higher ERC-FC in the DMN, ECN, and reward networks. This is consistent with previous literature, which has reported sex differences in structural and functional brain organization [42, 43]. The SN has been implicated in the detection and processing of salient stimuli and has been found to be affected in AD [44]. The DMN, which is associated with self-referential thinking and episodic memory, has been shown to be disrupted in AD, with females being more vulnerable to DMN alterations [45]. The ECN is involved in executive functions such as working memory, attention, and cognitive control, and its disruption has been linked to AD progression [46]. Lastly, the reward network, which includes the vmPFC and caudate, is associated with reward processing and decision-making, and its alterations have been reported in AD [47]. The functional annotation of the sex-specific ERC-FC network indicated that males CUOA-Aβ + mainly related behavioral terms of primary sensor and motor function, while females CUOA-Aβ + mainly related behavioral terms of higher cognitive function, such as social, mental states and value. Taken together, our findings underscore the notion that males and females employ distinct compensatory strategies in response to neuropathological alterations associated with Aβ accumulation.

The interaction between sex and APOE genotype in our study was found in the left PosC/SPL and left pCbl, suggesting that the effects of these factors on brain connectivity are not uniform across the brain. The PosC/SPL has been implicated in the processing of somatosensory information, and its disruption has been reported in AD [48]. The cerebellum has been increasingly recognized for its role in cognitive functions, and alterations in cerebellar connectivity have been observed in AD [49]. The observed interactions between sex and APOE genotype in these regions may reflect differential vulnerability to AD pathology, which may have implications for the development of targeted interventions for individuals at risk of AD.

Our study also demonstrated that ERC-FC was associated with executive function and memory performance in individuals with CUOA-Aβ + . These findings are in line with previous research, which has reported associations between functional brain connectivity and cognitive performance in preclinical AD [50]. The observed associations may reflect the impact of early AD pathology on the functional organization of the brain, which may subsequently lead to cognitive decline [51]. Understanding the relationship between brain connectivity and cognitive performance may provide valuable insights into the early detection of AD and inform the development of interventions to preserve cognitive function in at-risk individuals [2].

The strengths of this study include the large sample size, rigorous methodology, and comprehensive assessment of cognitive performance. However, there are some limitations worth noting. First, our study focused on cognitively normal individuals with Aβ + pathology, and the generalizability of our findings to other populations, such as individuals with mild cognitive impairment or dementia, is unclear. Second, the cross-sectional design of our study precludes the establishment of causal relationships between sex, APOE genotype, ERC-FC, and cognitive performance. Longitudinal studies are needed to investigate the temporal dynamics of these relationships and to determine whether the observed associations predict the progression to AD. Lastly, our study did not assess other potential modulators of ERC-FC, such as lifestyle factors, inflammatory markers, or other genetic variants, which may also contribute to the observed effects.

## Conclusion

In conclusion, the current study adds to our understanding of the relationship between sex, Aβ, and early brain function alteration in preclinical AD by examining sex differences in the ERC functional network in CUOA-Aβ + . These findings may contribute to our understanding of the mechanisms underlying sex differences in the early stages of AD and inform the development of personalized interventions to prevent or delay cognitive decline in preclinical AD.

## Data Availability

The A4 data are freely available to download after application (https://ida.loni.usc.edu/). The complete A4 Study Team list is available at a4study.org/a4-study-team.
